# A Carbon Trace Detection Method for Oil-Immersed Transformers Based on Superimposed Illumination Estimation and Multi-Scale Feature Fusion

**DOI:** 10.3390/s26134223

**Published:** 2026-07-03

**Authors:** Hongxin Ji, Zhennan Shi, Jiaqi Li, Xinghua Liu, Liqing Liu

**Affiliations:** 1 School of Electrical Engineering, China University of Mining and Technology, Xuzhou 221116, China; ts24230126p31@cumt.edu.cn (Z.S.); ts23230113p31@cumt.edu.cn (J.L.); 2 College of Mechanical and Electronic Engineering, Shandong Agricultural University, Tai’an 271018, China; 3 State Grid Tianjin Electric Power Research Institute, Tianjin 300180, China; liulq328@126.com

**Keywords:** oil-immersed transformer, discharge carbon trace, defect detection, attention mechanism, image enhancement, power robot

## Abstract

Accurately locating and reliably diagnosing insulation defects in oil-immersed transformers remains challenging. To overcome this, a micro-robot is employed to autonomously identify partial discharge (PD)-induced carbon traces on the insulation surface of the core components. Accurately capturing the multi-scale complex features of surface-discharge carbon traces under low-illumination conditions is critical for effective defect detection. Therefore, to address the obscurity of carbon trace features caused by insufficient illumination inside oil-immersed transformers, a Retinex-based image enhancement algorithm with superimposed illumination estimation is proposed. By transforming the original image into the HSI color space and integrating negative-image illumination fusion, this algorithm decouples brightness from chromaticity and preserves dark-region details, thereby reducing color distortion and enhancing carbon trace features. Furthermore, to handle the significant scale variations in carbon traces, a C2f module integrated with spatial and channel synergistic attention (SCSA) is designed. This module employs multi-scale depthwise separable convolutions and wide-channel self-attention to enhance cross-scale feature representation and reduce redundancy. Moreover, to address the feature resolution degradation in the fast spatial pyramid pooling module, which hinders the accurate perception of tiny carbon traces, a poly kernel inception atrous spatial pyramid pooling module (PKI-ASPP) is adopted. This preserves precise morphological details and minimizes the missed and false detection rates for tiny carbon traces. Finally, to tackle the difficulties in fusing complex morphological features, a deformable large kernel attention (DLKA) module is introduced into the neck network. This adapts to irregular carbon trace shapes, significantly improving the localization and learning of complex morphologies. Experiments on a transformer PD carbon trace dataset demonstrate that the proposed model significantly improves perceptual capabilities for carbon traces with massive scale variation. The improved model outperforms the baseline across all evaluation metrics, with mAP50 improved by 2.7% and mAP50-95 improved by 7.9%. These results indicate that the proposed method is highly reliable, providing solid technical support for internal surface discharge intensity detection and insulation condition assessment in oil-immersed transformer maintenance.

## 1. Introduction

Transformer insulation degradation represents the predominant cause of equipment failures, primarily occurring in longitudinal and main insulation structures. Therefore, regular monitoring of internal insulation integrity, supplemented by timely maintenance and repairs, is essential for ensuring power supply reliability and energy security. However, direct visual inspection within oil-filled sealed tanks remains highly challenging. Conventional non-electrical diagnostic approaches, such as dissolved gas analysis (DGA) [1,2,3] and infrared thermography [4], inherently lack precision for defect localization, fault classification, and severity assessment. Although recent machine-learning and residual-network-based diagnostic methods have improved transformer fault classification from monitoring data [5,6], they still provide indirect state information rather than direct visual evidence of insulation-surface defects. Standard inspection procedures, which involve oil drainage, metal casing disassembly (including lifting tank or core), or manual entry through manholes, serve as primary diagnostic tools for internal conditions. However, they suffer from substantial limitations, including complex protocols, significant safety hazards, high costs, heavy machinery requirements, operational risks and prolonged outages that compromise grid reliability and economic efficiency.

Transformer inspection micro-robots equipped with integrated propulsion modules demonstrate exceptional maneuverability and control precision in confined spaces [7]. By accessing oil-filled units through drain valves, these robots utilize visual sensors to inspect internal walls and critical components, enabling direct detection and progression assessment of insulation defects. Global research efforts have accelerated in this domain. In 2018, ABB pioneered the Txplore™ inspection robot [8], which received the 2019 Electrical Review Award for its innovative design. In 2020, Shenyang Ligong University developed a 19 cm spherical swimming inspection robot (SSTIR) featuring buoyancy-assisted jet propulsion systems, subsequently advancing hovering control algorithms [9] and visual localization techniques for floating robotic platforms [10]. Dual-loop robust control has also been investigated to improve the motion stability of inspection robots in oil-immersed transformers [11]. Moreover, collaborators from Tsinghua University, China University of Mining and Technology, and Tianjin Electric Power Research Institute have conducted multidimensional investigations encompassing oil-based positioning and attitude estimation [12,13,14], inspection path planning [15], and defect recognition [16,17,18,19,20,21].

During inspection operations, the images captured by the robot’s visual sensors are transmitted via wireless signals and stored in computers, providing critical references for defect identification and maintenance documentation. Nevertheless, extensive inspection coverage generates large-scale datasets characterized by suboptimal image quality, complex backgrounds and significant morphological variations in carbon traces. Relying solely on manual assessment to judge the degree of insulation degradation is not only inefficient and labor-intensive but also highly susceptible to missed detections and erroneous judgments. Therefore, the accurate, efficient, and real-time extraction of internal defect information is a critical prerequisite for successfully executing autonomous robot inspection tasks.

Deep learning-based carbon trace detection offers advantages in feature extraction efficiency and analytical reliability, contributing to the evaluation of discharge development. Two-stage detection models, represented by the Faster R-CNN model, operate through initial region proposal generation followed by position refinement and classification. While they achieve high accuracy and reliable results, their computational complexity and long detection times make them unsuitable for field deployment [22].

End-to-end transformer-based object detection algorithms such as DETR, deformable DETR, and RT-DETR eliminate NMS, enabling a fast detection process [23,24,25]. However, they suffer from long training cycles and high hardware configuration requirements, making them impractical for portable detection terminals. By contrast, one-stage detection algorithms represented by YOLO [26] and SSD [27] transform object detection into a regression framework. Through convolutional neural network downsampling, they directly output target classes and predicted bounding box locations, effectively balancing detection accuracy and speed, thus meeting the deployment requirements of maintenance scenarios.

Considering the unique characteristics of carbon trace morphology, PD initially manifests as minute dot-like formations or discrete clusters on oil-paper insulation surfaces. If insulation conditions remain unimproved, these progress to surface creepage discharge across the oil-paper insulation. During this advanced stage, carbon tracks develop dendritic structures, extending directionally with significant size variations, labyrinthine edge morphology, and intricate microstructures at the carbonized branch tips [28,29,30,31,32].

To meet the demand for the accurate and efficient identification of internal transformer carbon traces, this study proposes an improved defect detection method. First, to mitigate the poor image visibility caused by low-illumination conditions inside oil-immersed transformers, a Retinex-based image enhancement algorithm with superimposed illumination estimation is proposed. By transforming the original image into the HSI color space and constructing a Laplacian pyramid, this algorithm achieves brightness–chromaticity decoupling and enhances high-frequency information, thereby reducing color distortion and strengthening carbon trace feature details. Second, to handle the significant scale variations in carbon traces during discharge progression, a C2f module integrated with an SCSA module is designed. It employs large-scale depthwise separable convolutions and wide-channel self-attention to enhance multi-scale feature representation and reduce redundant features. Third, to prevent the loss of fine-grained spatial details inherent in the SPPF module—which often blurs the morphology of tiny carbon traces—a PKI-ASPP module is adopted. By combining multi-scale depthwise separable convolutions with atrous convolutions, it preserves richer morphological and detail features than standard pooling, thereby reducing the missed and false detection rates for tiny and complex carbon traces. Finally, to tackle the difficulties in fusing complex carbon trace morphology and multi-scale features, a DLKA module is introduced in the neck network. Utilizing deformable convolutions with large kernels, it expands the receptive field and adapts to irregular carbon trace shapes, significantly aiding the model in localizing tiny traces and learning complex morphologies.

## 2. Transformer Internal Inspection Robot

### 2.1. Transformer Internal Inspection Robot Structure

**Figure 1:**
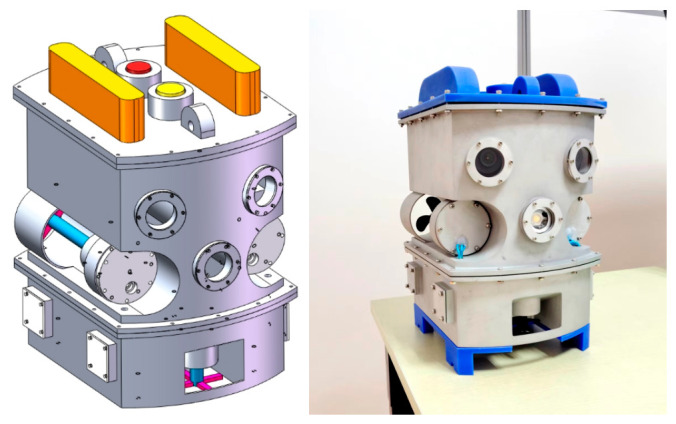
Transformer internal inspection robot structure.

The transformer internal inspection robot used in this study has a square body. And a curved housing is adopted in the horizontal forward and backward directions to reduce motion resistance and improve inspection time. Its overall structure is shown in Figure 1.

Two horizontal propulsion devices are installed on the middle housing of the internal inspection robot to realize flexible horizontal motion. Two vertical propulsion devices are installed at the bottom to realize vertical motion. To balance inspection efficiency and imaging quality, the maximum cruising speed is set to 10 cm/s, and the maximum ascending and descending speed can reach 6 cm/s. Gravitational variation and buoyancy compensation techniques are adopted to not only reduce power consumption but also improve attitude stability, extending the cruising endurance to 50 min.

Because the transformer is completely sealed by a metallic shell, filled with transformer oil, conventional localization methods such as LiDAR, visual cameras, and GPS have difficulty in achieving precise localization inside the transformer. Ultrasonic localization does not require illumination, has a wide detection range in transformer oil, and offers high positioning accuracy. Then an ultrasonic localization array is used to achieve spatial positioning of the inspection robot. The positioning accuracy is within 5 cm, meeting the application requirements of transformer robots.

The visual perception system of the transformer internal inspection robot is located in three sealed transparent windows of the inspection robot. A high-brightness wide-area light is installed in the lower window. The two upper windows are equipped with a high-resolution camera (C3_ISC3NA) to transmit the transformer’s internal images. The binocular camera lenses have a 30-degree viewing-angle difference, which improves the robot’s detection view field.

### 2.2. Portable Operation Terminal and Defects Detection Software for the Inspection Robot

**Figure 2:**
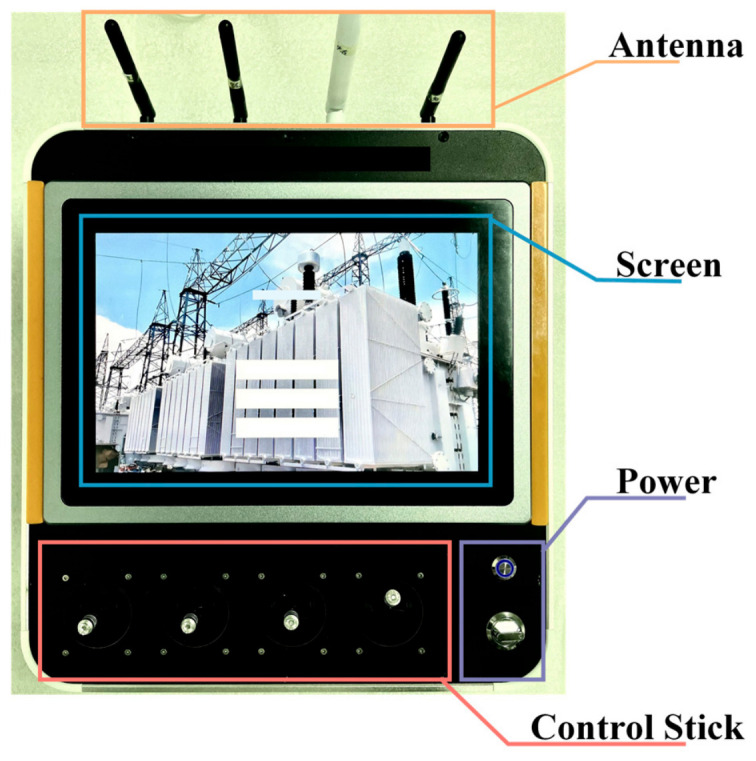
Portable operation terminal for the inspection robot.

The portable operation terminal for the inspection robot serves as a hardware interface to remotely control the robot’s movement and internal defect detection. It integrates three functional modules: wireless communication for robotic signals, manual joystick controls, and visual inspection processing for transformer defects. The terminal’s architecture is depicted in Figure 2.

The defect inspection software is deployed on the portable operation terminal, as illustrated in Figure 2. It utilizes Python’s PySide6 framework for GUI development, incorporating login panels, functional selection menus, and task execution interfaces. The proposed defect inspection algorithm uses a deep learning framework to realize three tasks: image enhancement of carbon trace, object detection of internal defects and instance segmentation of internal defects. The developed defect inspection software for the inspection robot has the advantages of user-friendly operation, comprehensive functionality, and strong extensibility. It serves as a practical platform for conducting internal defect detection tasks.

## 3. Partial Discharge Carbon Trace Dataset

### 3.1. Acquisition Platform for Partial Discharge Carbon Trace

To address scarce training data for detection tasks, this study established an experimental platform to reproduce intense normal electric fields across oil-paper insulation systems. Through systematic observation of carbon deposition dynamics and defect morphology replication, a comprehensive carbon trace database was constructed.

**Figure 3:**
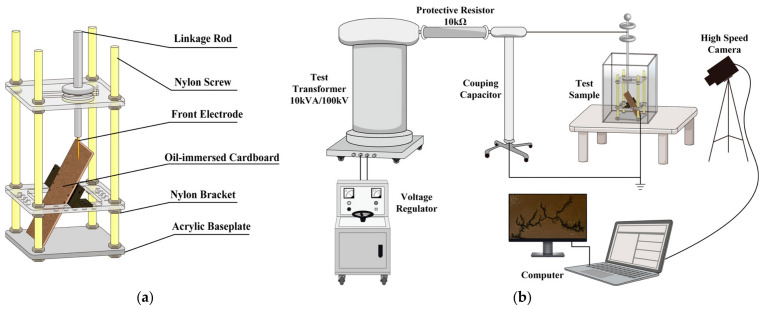
Acquisition platform for partial discharge carbon trace: (**a**) needle-plate discharge model; (**b**) the whole diagram of the experimental platform.

As shown in Figure 3, the platform comprises two main components: an oil-paper insulation needle-plate discharge subsystem and a carbon trace capture subsystem. As shown in Figure 3a, the needle-plate electrode configuration features a tungsten needle with a 0.2 mm curvature radius secured by brass front-end fixtures, paired with a standard transformer pressboard measuring 25 cm × 15 cm mounted by adjustable polyamide supports on an acrylic baseplate. Auxiliary structures include nylon adjustment screws, a grading ring, and a linkage rod. As shown in Figure 3b, the discharge subsystem is put into a transparent acrylic test chamber, which is filled with Karamay #25 transformer oil. Powered by an SB-10 KVA/100 KV AC testing transformer, the imaging subsystem employs a high-speed camera (HTSUA134GC/M, 1.3 million pixels, 211 FPS) to transmit carbon trace images via an HDMI interface. This platform enables systematic investigation of carbon trace morphology under different operational states through adjusting operational parameters including dielectric tilt angle, electrode gap distance, and discharge characteristics.

**Figure 4:**
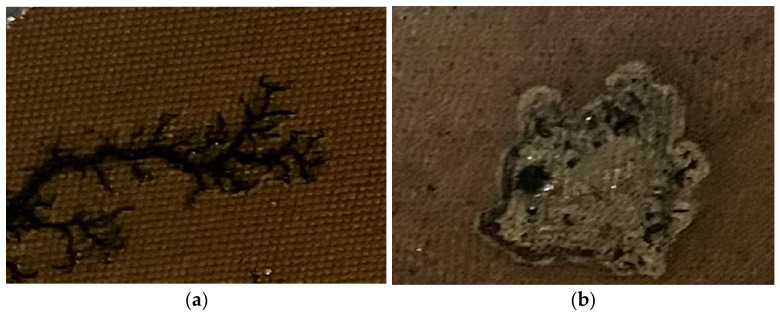
Partial discharge carbon traces: (**a**) dendritic carbon traces; (**b**) clumpy carbon traces.

Two typical categories of discharge carbon trace images, namely dendritic and clumpy carbon traces, were finally obtained, as shown in Figure 4. Due to the difficult formation of carbon traces, the amount of carbon trace samples collected in simulation experiments was still insufficient for deep learning model training. To expand the sample amount, the original samples generated by discharge experiments were first divided into training, validation, and test sets. Data augmentation was then performed independently only within each dataset, ensuring that similar samples come from the same original discharge experiment and their augmented images would not appear in different datasets.

During dataset construction, a more rigorous splitting strategy was adopted. The 654 original images were obtained from different discharge experiments, and 206 images (116 dendritic and 90 clumpy) were selected as the test set. The remaining images were split at an 8:2 ratio into a training set (359 images) and a validation set (89 images). After LabelImg annotation and illumination adjustment, random rotation, mirroring, and stretching were applied to the remaining carbon trace images in the training and validation sets for data augmentation. A complete training set containing 3658 images (2329 dendritic and 1329 clumpy images) was finally constructed, providing reliable data support for model training and evaluation.

### 3.2. Retinex with Superimposed Illumination Estimation for Transformer Image Enhancement

Image acquisition inside sealed, oil-filled transformers is challenging. The lack of natural illumination requires fill-light illumination. And insulating oil absorbs and scatters light, causing color distortion, blurred details, low contrast, and uneven brightness [33,34]. In addition, slight differences in the robot’s hovering position affect the spatial relationship between the lens and the carbon traces. The complex internal structure of the transformer may also block the fill light, producing shadows on inspected surfaces or obscuring detection targets. These problems will reduce the quality of carbon trace images and impair the reliability of insulation defect diagnosis. Therefore, this study investigates imaging enhancement algorithms inside the transformer oil.

Researchers have investigated low-light image enhancement mainly through two technical routes: traditional methods and deep learning methods. Traditional methods rely on physical models or image statistical properties. They improve image color, brightness, and contrast by inverting degradation processes or adjusting pixel values. However, such methods have difficulty for uneven illumination correct and noise suppression in complex scenes [35]. In recent years, because of stronger modeling capability and multi-scale feature representation, deep-learning-based image enhancement methods have become a research focus. Supervised methods establish nonlinear mappings between low-quality and high-quality images, thereby reducing problems such as inaccurate illumination estimation, artifact generation, and noise amplification. However, supervised methods rely on paired normally illuminated images, limiting their application scenarios and increasing engineering difficulty [36]. Unsupervised methods eliminate dataset construction barriers, but they lack explicit supervision and often ignore physical characteristics such as the application scenario and degradation mechanism. Therefore, their enhancement results may be random and may not accurately match human-specified task objectives [37]. In addition, diffusion-model-based enhancement methods mainly learn uncertain mapping between low-light and normally exposed images and consider more complex degradation modeling and detail reconstruction [38,39], but they usually cannot meet the real-time requirements of industrial visual detection.

**Figure 5:**
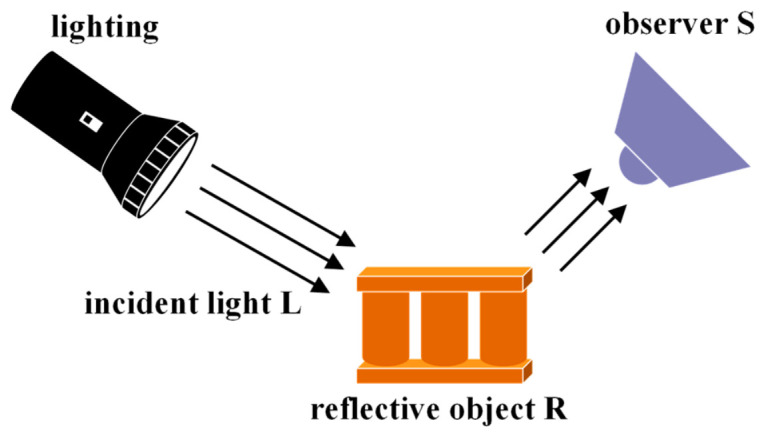
Retinex theory model diagram.

To process submerged carbon trace images, we designed a low-light image enhancement algorithm based on the Retinex theory. This approach draws upon color constancy theory to achieve more natural-looking image enhancements. A schematic overview of its operational principle is provided in Figure 5.

This theory assumes that an impaired original image $S\left(x,y\right)$ results from the synthesis of two components: an illuminance component $L\left(x,y\right)$ induced by a light source and an object-specific reflection component $R\left(x,y\right)$, expressed as follows.

(1) $$S\left(x,y\right)=R\left(x,y\right)\cdot L\left(x,y\right)$$

**Figure 6:**
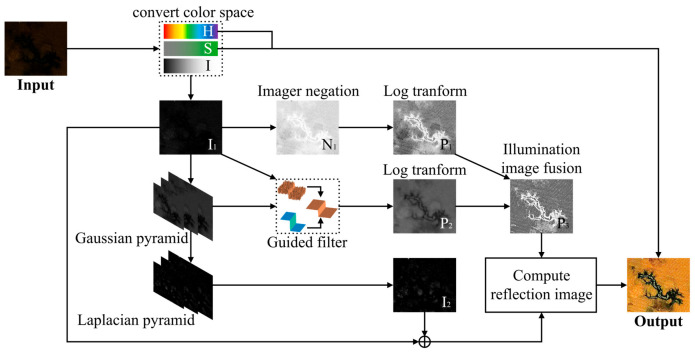
The improved Retinex algorithm integrated with superimposed illumination estimation.

As human perception of surface color characteristics in visible objects primarily relies on reflected information from object surfaces, the MSRCR algorithm has been proposed [40]. However, while enhancing overall brightness, the MSRCR algorithm excessively intensifies dark-region details, causing the intricate edges blurred and color deviation with respect to carbon traces. To address this limitation, we proposed the improved Retinex algorithm integrated with superimposed illumination estimation for image enhancement, as shown in Figure 6. By integrating negative images with multi-scale illumination maps into composite estimates, it suppresses over-enhancement in extremely dark regions, preserving vital carbon trace characteristics.

Initially, the input image is transformed from RGB color space to HSI color space to achieve decoupling of intensity and chrominance. The conversion process from R, G, B channels to H, S, I channels is expressed as follows:

(2) $$\left\{\begin{array}{c} H=\left\{\begin{array}{c} \theta \\ 2\pi -\theta \end{array}\right.\\ \theta ={cos}^{-1}\left[\frac{\frac{1}{2}\left[\left(R-G\right)+\left(R-B\right)\right]}{\sqrt{{\left(R-G\right)}^{2}+(R-B)(G-B)}}\right] \end{array}\right.$$

(3) $$S=1-\frac{2min(R,G,B)}{R+G+B}$$

(4) $$I=\frac{1}{3}(R+G+B)$$

In the final stage of the algorithm, to prevent color shifts and distortions arising from applying image enhancement directly to the original RGB channels, the corrected intensity channels with preserved hue and saturation components are recombined. Furthermore, selectively enhancing the intensity component significantly reduces computational complexity while accelerating image processing speed.

The proposed method employs a Laplacian pyramid to enhance high-frequency image information. It first applies Gaussian filters $g\left(x,y\right)$ interatively to downsample the original image to construct a Gaussian pyramid, where the level l-th image is denoted as $G_{l}\left(i,j\right)$, expressed as follows:

(5) $$G_{l}\left(i,j\right)=\sum_{x=-2}^{2}\sum_{y=-2}^{2}g\left(x,y\right)G_{l-1}\left(2i+x,2j+y\right)$$

The Laplacian pyramid decomposes the high-frequency details into multiple distinct frequency bands. Let ${Laplace}_{i}$ denote the level i in the Laplacian pyramid, which is constructed using Formula (6):

(6) $${Laplace}_{i}=G_{i}-upsample\left(G_{i+1}\right)$$

As each level i of the Laplacian pyramid is constructed by subtracting the upsampled level i+1 of the Gaussian pyramid from its level i counterpart, it retains multi-scale high-frequency details at the corresponding position in the original image, thereby enabling effective extraction of carbon trace characteristics.

In the brightness enhancement section, a guided filtering method was adopted to refine multi-scale illumination estimations. First, each layer of the Gaussian pyramid is converted into an illumination estimation $U^{p}\left(p\in 1,2,3,\cdots ,l\right)$ with uniform dimensions. Subsequently, guided filtering is applied to produce edge-preserving and more accurate illumination estimation. As a linear shift-invariant filtering process, the proposed algorithm utilizes the original image’s intensity channel $I_{1}$ as the guidance image, while upsampling each Gaussian pyramid layer to match the resolution of the source image as input $U^{p}$. For any pixel i in the output image L, its value is computed according to:

(7) $$L_{i}=a_{k}U_{i}^{p}+b_{k},\forall i\in \omega_{k}$$

where $U_{i}^{p}$ represents the value of pixel i in the output image, $\omega_{k}$ represents the local window encompassing pixel k; $a_{k}$ and $b_{k}$ are linear coefficients within this local window, determined by an objective function expressed as:

(8) $$E\left(a_{k},b_{k}\right)=\underset{\left(a_{k},b_{k}\right)}{\min}\sum_{i\in \omega_{k}}\left({\left(I_{1}-\left(a_{k}U_{i}^{p}+b_{k}\right)\right)}^{2}+\lambda a_{k}^{2}\right)$$

As image dark regions contain richer target information, processing negative images provides more advantages for carbon trace identification. A negative image is created by inverting the brightness values of the input image, mathematically expressed as:

(9) $$P_{1}=lg\left(1-I_{1}\right)$$

Performing illumination fusion on the weighted multi-scale illumination estimation $N_{1}$ and negative images $N_{2}$, while incorporating two weight factors α=9 and β=0.8 to regulate the synthesis ratio, the final illumination estimation $P_{3}$ is yielded, expressed as:

(10) $$P_{3}\left(x,y\right)=max\left(\alpha P_{1}\left(x,y\right),\beta P_{2}\left(x,y\right)\right)$$

$R\left(x,y\right)$ is calculated as the enhanced image expression according to Retinex theory as follows:

(11) $$lg\left(R\left(x,y\right)\right)=\left(lg\left(I_{1}\right)+lg\left(I_{2}\right)\right)-\gamma P_{3}$$

where γ is introduced to globally control the magnitude of image brightness elevation. Through multiple adjustment tests, it was found that γ=0.85 achieved an ideal balance between visual enhancement and defect-detail preservation. It is suitable for transformer internal carbon trace image enhancement.

**Figure 7:**
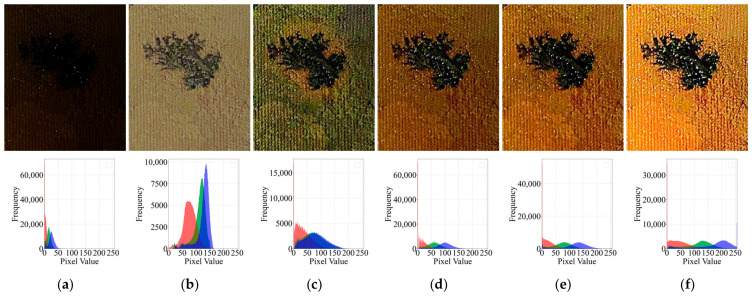
Comparison of image enhancement: (**a**) original image, (**b**) MSRCR algorithm, (**c**) CLAHE algorithm, (**d**) SRIE algorithm, (**e**) DCP algorithm, (**f**) ours.

To validate the suitability of the proposed Retinex with superimposed illumination estimation for carbon trace image enhancement, this study conducts comparative experiments using multiple distinct enhancement algorithms. The visual comparison results against primary methods including MSRCR, SRIE and DCP algorithms are shown in Figure 7.

As shown in Figure 7, the original image has low overall brightness, making carbon traces difficult to distinguish from the background. After MSRCR processing, the sample’s brightness and contrast are improved, but obvious color distortion is observed, and carbon trace details are blurred with considerable feature loss. After CLAHE processing, brightness and contrast are further improved, which helps to preserve carbon trace features, but the output image shows severe color cast and noise amplification. The SRIE algorithm moderately improves the sample’s brightness while preserving color, improving the natural visual appearance. The DCP algorithm also improves brightness and contrast while maintaining color restoration, but local dark-region carbon trace details are blurred and lost. The proposed algorithm controls pixel stretching in extremely dark regions while expanding the pixel distribution range, improving overall brightness, reducing color distortion, and strengthening carbon trace details. It facilitates subsequent target feature learning by the carbon trace recognition network.

The innovation of the proposed Retinex-based image enhancement algorithm with superimposed illumination estimation is mainly reflected in the following aspects. First, considering the color contrast characteristics of typical carbon traces, this study combines Retinex-based illumination estimation with HSI channel separation. This transformation simplifies illumination correction and reduces subsequent processing complexity, while improving image brightness and preserving the color relationship between carbon traces and the oil background as much as possible. Thus, it could reduce color distortion in common methods such as MSRCR.

Second, for the complex fine-branch morphology of carbon traces, a Laplacian pyramid is used to efficiently extract high-frequency texture information from typical carbon trace images. The image is decomposed into detail information at different resolution levels, so that carbon trace contours and tiny local carbon trace textures maintain good structural continuity.

Third, a guided filtering method is used to correct multi-scale illumination estimation, giving the illumination component edge-preserving property and reducing boundary blur caused by traditional smoothing filters.

Fourth, a negative-image superimposed illumination estimation is introduced. By fusing dark-region information, it enhances dark-detail features of carbon traces and suppresses noise amplification and pseudo-texture problems caused by excessive stretch of extremely dark regions.

Fifth, the proposed method has strong interpretability and low algorithmic complexity, making it suitable to embed in the image acquisition and defect detection workflow of transformer internal inspection robots. It does not require construction of a large-scale paired low-light training dataset and avoids the domain generalization risk of deep enhancement networks in industrial small-sample scenarios. The proposed low-light enhancement algorithm for carbon trace images provides high-quality input data for subsequent carbon trace defect detection training and inference, thereby improving insulation defect detection accuracy.

## 4. Improvement of Carbon Trace Detection Algorithm Based on Multi-Scale Feature Fusion

### 4.1. The Improved Detection Model Based on YOLOv10

Two-stage detection models represented by Faster R-CNN generate candidate regions and then refine positions and identify categories, achieving high and reliable detection accuracy. However, such models are computationally complex and time-consuming, making them difficult to use in maintenance scenarios. Transformer-based end-to-end object detection algorithms eliminate NMS and can achieve an efficient end-to-end detection process, but they still suffer from long training cycles and high computation requirements and are not suitable for portable detection terminals.

YOLOv10 is adopted as the baseline model for transformer internal carbon trace detection [41]. This model is designed for very low latency and end-to-end real-time detection. The backbone is responsible for extracting both shallow and deep features from input images with maximal richness. Employing an FPN-PAN structure in the neck enables multi-scale feature fusion of carbon traces across different layers, thereby enhancing semantic representation and localization capabilities.

Compared with previous YOLO models, YOLOv10 was comprehensively optimized. In the backbone and neck networks, SCDown with spatial and channel decoupled downsampling is used to optimize the model structure and improve real-time performance. The detection head incorporates a lightweight classification module that eliminates redundant NMS operations. While maintaining high detection accuracy, this reduces dependence on post-processing during inference and helps to lower overall inference latency.

**Figure 8:**
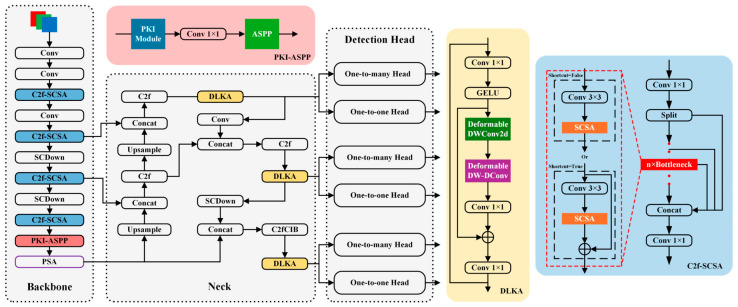
Improved detection model structure of internal carbon trace.

The acquisition of partial discharge carbon traces in the transformer presents several distinctive characteristics: limited lighting conditions inside transformers lead to blurred imaging of carbon trace; carbon traces formed during initial discharge stages exhibit extremely small dimensions; during discharge development stages, these traces progressively expand into dendritic structures extensively covering insulating paper barrier surfaces. To address these challenges in carbon trace acquisition, this paper implements optimized improvements on the classical object detection method and proposes a dedicated model for detecting discharge carbon traces in transformers, the architecture of which is illustrated in Figure 8.

### 4.2. Improved Backbone Based on Spatial and Channel Synergistic Attention Module

The transformer’s internal structure exhibits intricate complexity with numerous unknown textures and edge geometries, while carbon traces display critical characteristics including high complexity and multi-scale variations. However, employing a C2f block with fixed and limited receptive fields in the backbone hinders the model’s ability to accurately and efficiently extract multi-scale edge information, textural features, and enhanced contour and shape representation from carbon trace images. When the typical object detection method fails to sufficiently capture carbon trace characteristics, similar ambiguous background patterns adversely impact performance, ultimately constraining their effectiveness in cluttered scenarios.

**Figure 9:**
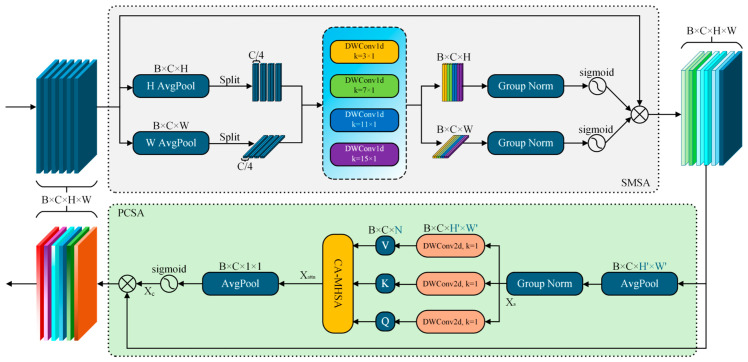
Mixed attention module structure of SCSA.

Addressing these challenges inherent to the detection method of carbon tracing in transformers, this paper proposes the SCSA module to enhance Bottleneck components within C2f units, constructing a lightweight architecture named C2f-SCSA, as depicted in Figure 9.

The design employs a shareable multi-semantic spatial attention module (SMSA) to boost multi-scale feature extraction capabilities, followed by a progressive channelwise self-attention module (PCSA) that mitigates information disparities across receptive fields [42]. SMSA and PCSA are serially connected with residual connections to preserve crucial features extracted by convolutional networks.

(12) $$X_{out}=SCSA\left(X_{in}\right)=PCSA\left(SMSA\left(X_{in}\right)\right)$$

The construction of the SMSA module is inspired by the coordinate attention module (CA) [43]. First, it performs stripewise average pooling along both the height and width dimensions of the input feature map, generating two sets of 1D features to precisely capture positional attention cues of carbon traces. Subsequently, each set is evenly divided into four sub-features, which enables multi-scale 1D depth separable convolution to extract carbon trace characteristics, expressed as:

(13) $${\hat{X}}_{H}^{i}={DWConv1d}_{n_{i}}\left(X_{H}^{i}\right)$$

(14) $${\hat{X}}_{W}^{i}={DWConv1d}_{n_{i}}\left(X_{W}^{i}\right)$$

where $X_{H}^{i}$ and $X_{W}^{i}$ represent the sub-feature i of the input horizontal and vertical 1D feature sets, respectively, while $n_{i}$ denotes the convolutional kernel applied to the sub-feature i.

PCSA utilizes a channelwise multi-head self-attention module (CA-MHSA) to integrate multi-scale spatial information generated by the SMSA. PCSA omits channel compression, prevents loss of critical features, and facilitates formation of a comprehensive and rich feature representation. It first applies local average pooling combined with group normalization to produce downsampled spatial tokens, effectively reducing the computational complexity of the self-attention mechanism. Subsequently, 2D depth separable convolution is employed for efficient extraction and computation of per-channel self-attention weights, expressed as:

(15) $$F={DWConv2d}_{\left(1,1\right)}$$

(16) $$\left\{\begin{array}{c} Q=F^{Q}\left(X_{a}\right)\\ K=F^{K}\left(X_{a}\right)\\ V=F^{V}\left(X_{a}\right) \end{array}\right.$$

(17) $$X_{attn}=Attn\left(Q,K,V\right)=Softmax\left(\frac{QK^{T}}{\sqrt{C}}\right)*V$$

(18) $$X_{c}=\sigma \left({AvgPool}_{\left(1,1\right)}\left(X_{attn}\right)\right)$$

The proposed C2f-SCSA module demonstrates superior performance in processing carbon traces with intricate details and small-scale targets, effectively enhancing the model’s capability to extract edge patterns, textures, colors, and other fine-grained features. It substantially reduces computational complexity while strengthening feature representation at critical scales. In addition, it suppresses redundant information, and improves target feature capture accuracy. This provides precisely localized key feature information for subsequent feature fusion in the neck.

### 4.3. Improved Atrous Spatial Pyramid Pooling Module Based on Poly Kernel Inception

The classical object detection method employs SPPF module to integrate multi-scale features and positional information of carbon traces [44], achieving hierarchical representation through parallel pooling operations. However, due to extreme scale variations and intricate details of internal transformer carbon tracing, repeated max pooling operations discard substantial localized characteristics while blurring edge information. Therefore, it results that the SPPF module fails to accurately represent complex carbon trace features and compromises feature expression levels in the neck part.

**Figure 10:**
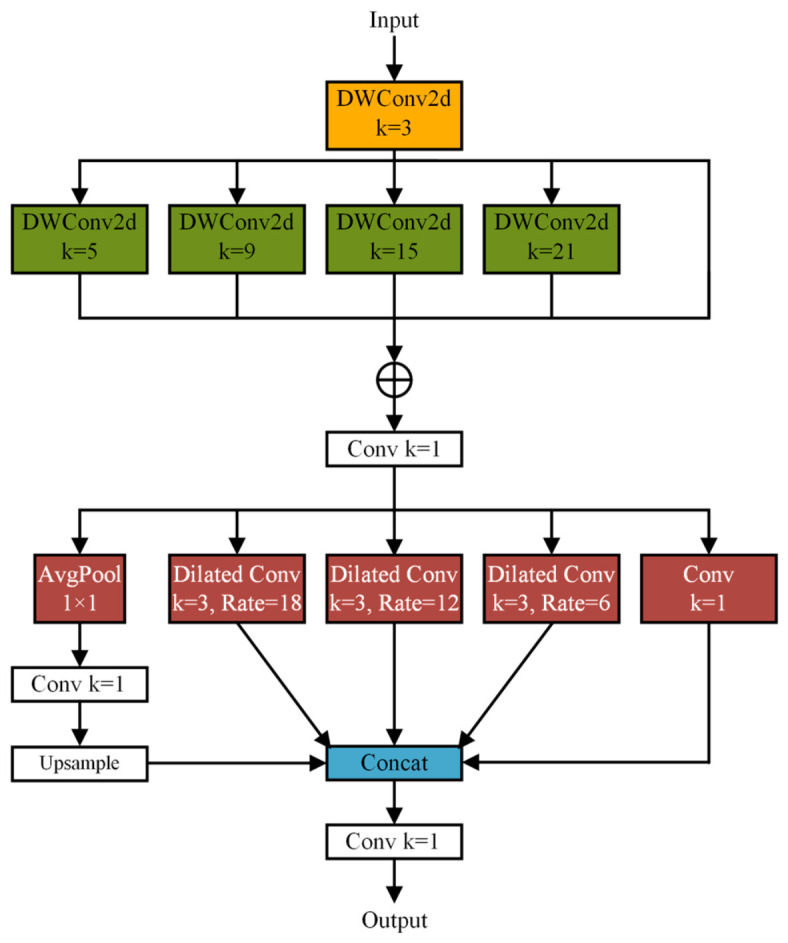
PKI-ASPP module structure.

Therefore, we proposed an improved ASPP module named PKI-ASPP, utilizing dilated convolutions with varying expansion rates across multiple scales to extract image features, supplemented by global average pooling for capturing long-range contextual relationships [45]. This approach effectively mitigates the spatial resolution degradation of the downsampling operation. Meanwhile, a PKI module is designed to assist dilated convolution operations in achieving larger receptive fields while mitigating sparse sampling issues [46]. The architecture of PKI-ASPP is illustrated in Figure 10.

The PKI module comprises depthwise separable convolutions with kernel sizes of 5, 9, 15, and 21. After extracting multi-scale features, these are superimposed and then fused using 1×1 convolutional kernels to integrate feature information from multi-scale receptive fields across different channels.

(19) $$P={DWConv2d}_{\left(3,3\right)}\left(X_{in}\right)$$

(20) $$Q^{n}={DWConv2d}_{\left(k^{n},k^{n}\right)}\left(P\right),n=1,\cdots ,4$$

(21) $$X_{out}={Conv}_{\left(1,1\right)}\left(P+\sum_{m=1}^{4}Q^{n}\right)$$

The proposed method effectively achieves complementarity between dilated convolution and multi-scale depthwise separable convolution in terms of receptive field coverage and multi-dimensional feature extraction capabilities, thereby reducing missed detection rates for both microscopic and complex carbon traces.

### 4.4. Improved Neck Based on Deformable Large Kernel Attention Module

**Figure 11:**
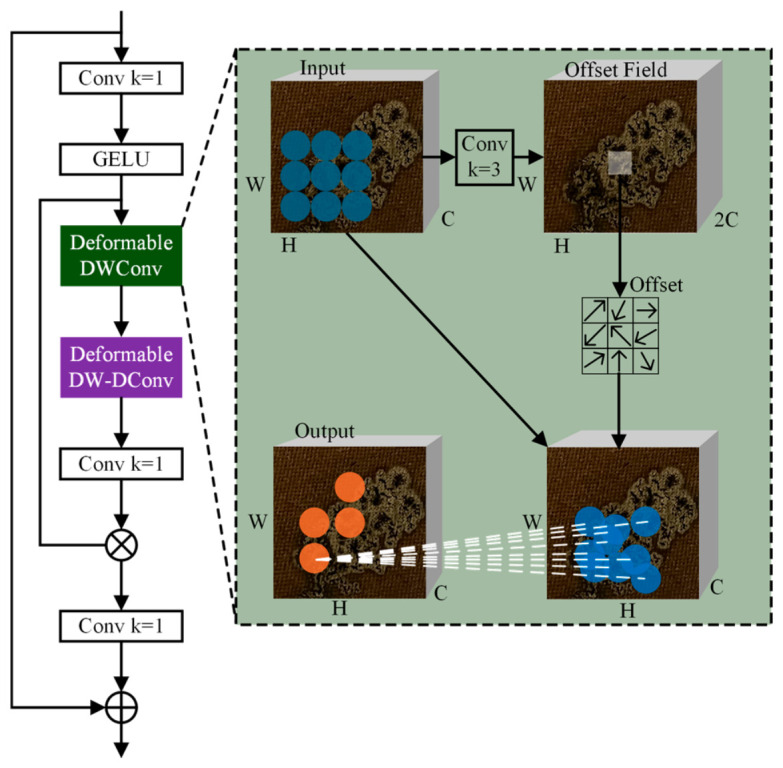
DLKA module structure.

Due to the presence of microscopic edges, textural patterns, and significant morphological variations in carbon traces in the transformer, conventional convolutional operations seriously constrain the shape and scope of receptive fields, due to which it samples exclusively within fixed 3 × 3 grids centered at output locations. This limitation impedes precise learning of complex carbon trace characteristics, causing spatial misalignment of critical positions and loss of fine details during feature fusion processes, ultimately resulting in suboptimal integration performance. To address these challenges, this paper introduces the DLKA module between the neck and detection head [47]. The module aims to enhance the neck’s capabilities in processing, fusing, and augmenting multi-level features, whose structure is illustrated in Figure 11.

The DLKA module achieves dual advancements: first, leveraging a large kernel attention module (LKA) through depthwise separable convolutions to expand receptive field dimensions, thereby enhancing the neck’s capacity for integrating deep semantic information and more effectively capturing global characteristics of large-scale targets. Second, incorporating deformable convolutions enables adaptively shaped receptive fields. It flexibly accommodates complex structural patterns, which could address challenges such as deformation, rotation, scale variations, and occlusion encountered with carbon traces. It successfully improves multi-scale feature fusion accuracy and anchor box localization precision. To counteract increased computational load from deformable operations, depthwise separable dilated convolutions combined with attention modules strike an optimal balance between complexity control and long-range contextual awareness. The mathematical formulation of the DLKA module is expressed as:

(22) $$I^{\prime }=GELU\left({Conv}_{\left(1,1\right)}\left(I\right)\right)$$

(23) $$I_{attn}^{\prime }={Conv}_{\left(1,1\right)}\left({Conv}_{DDW}\left({Conv}_{DDW-D}\left(I^{\prime }\right)\right)\right)$$

(24) $$Output={Conv}_{\left(1,1\right)}\left(I_{attn}^{\prime }\times I^{\prime }\right)+I$$

where $I\in R^{C\times H\times W}$ represents the input image and ${Conv}_{DDW}$ represents deformable depthwise separable dilated convolution. This paper employs a 5×5 ${Conv}_{DDW}$ and a 5×5 ${Conv}_{DDW-D}$ with a dilated rate of 3 to achieve optimal convolutional dimensions.

The introduction of learnable offsets is the key for enabling adaptive receptive field adjustment in deformable convolutions. If $p_{n}$ denotes the spatial offset of each sampling point relative to the center within a 3×3 convolutional kernel, $∆p_{n}$ represents the ideal sampling point’s deviation from the center. $\omega \left(p_{n}\right)$ signifies the weight assigned to the corresponding position in the kernel, $x\left(p_{0}+p_{n}\right)$ indicates the element value at position $p_{0}+p_{n}$ on the input feature map, and $y\left(p_{0}\right)$ represents the element value at the central position on the output feature map, then the expression for deformable convolution is formulated as:

(25) $$y\left(p_{0}\right)=\sum_{p_{n}\in R}\omega \left(p_{n}\right)*x\left({p_{0}+p}_{n}+∆p_{n}\right)$$

The proposed method endows the model’s neck with large-scale adaptively transformable receptive fields, significantly enhancing the detection accuracy of carbon trace detection systems. This advancement enables superior performance in tasks involving deformation handling and cluttered background scenarios.

## 5. Validation and Discussion of Carbon Trace Detection Model

### 5.1. Performance Evaluation Index

To accurately quantify the performance of the transformer internal carbon trace detection algorithm and verify the innovation and engineering practicality of the proposed method, appropriate evaluation metrics must be selected. Commonly used metrics for image detection models include precision (P), recall (R), accuracy, average precision (AP), mean average precision (mAP), number of parameters, and FPS. Subsequent sections will introduce the definitions and evaluative roles of these performance metrics in detail.

The P metric represents the proportion of true positive cases among all predicted positive instances, while the R metric indicates the proportion of true positive cases relative to all actual positive instances. Both metrics are computed by comparing recognition results against ground truth labels. Specifically, P evaluates what fraction of model-identified positive samples are genuinely positive, whereas R measures what portion of all actual positive samples were successfully identified by the model. Their mathematical formulations are as follows:

(26) $$P=\frac{TP}{TP+FP}$$

(27) $$R=\frac{TP}{TP+FN}$$

where TP represents true positives, FP represents false positives, FN represents false negatives, and TN represents true negatives. Consequently, an increase in precision signifies the enhanced capability of the model to distinguish between non-target and target objects, while improved recall indicates its strengthened ability to detect targets and minimize missed detections.

The AP metric builds upon the concepts of precision and recall. Since both precision and recall always fall within the range between 0 and 1, consequently the AP value also lies between 0 and 1. For each category, it is calculated as the area under the precision–recall (PR) curve, with the formulation shown below:

(28) $$AP=\int_{0}^{1}p\left(r\right)dr$$

The mAP metric is derived by computing the AP for each individual category and then calculating the average of these AP values across all categories. It serves as the most commonly used comprehensive evaluation metric in object detection methods. The mathematical formulation for mAP is expressed as:

(29) $$mAP=\frac{1}{N}\sum_{i=1}^{N}{AP}_{i}$$

where N represents the number of classification labels in the detection algorithm. The mAP metric can be further decomposed into mAP50 and mAP50-95 metrics: mAP50 denotes the average detection accuracy across all classes when using an IoU threshold of 50%, whereas mAP50-95 calculates the mean detection accuracy across all IoU thresholds ranging from 50% to 95% in increments of 0.05. As the optimal benchmark for evaluating object detection models’ overall performance, mAP50-95 provides a more holistic assessment than standalone mAP50, with higher values indicating superior robustness and consistent performance across diverse localization precision requirements.

Parameters is obtained by counting the parameters contained in the model’s convolutional layers, fully connected layers, output layers, and other structures, reflecting the model’s computational-resource requirements and training-data efficiency. FPS denotes frames per second, namely the amount of image information processed for storage and display per second. In computer vision tasks such as object detection, it is usually required that FPS is greater than 30 for real-time processing.

### 5.2. Platform Parameters for Model Training

To validate the enhancement effects of the improved detection method of carbon trace proposed in this paper, an experimental platform was established for model construction, training, and comparative verification. The configuration details of the training environment and parameters are listed in Table 1.

**Figure 12:**
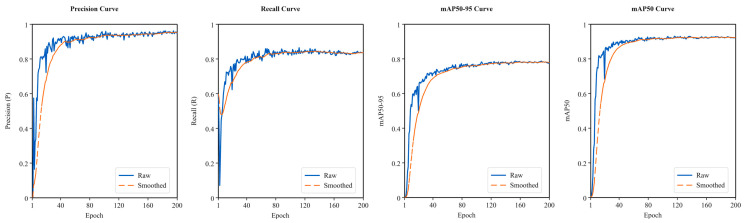
Detection algorithm training process.

The detection method of carbon tracing proposed in this paper was trained on a PD carbon trace dataset. As shown in Figure 12, both the localization loss and classification loss functions for the training and validation sets, along with confidence loss metrics, accuracy, recall rate, and average precision, have reached converged states.

To objectively assess the computational resources and training time required by the proposed method, we used the same dataset partition and number of training epochs and ensured that all comparison models were trained on the same computing platform with identical training configurations. The baseline model required approximately 6.5 h to complete one full training run, whereas the proposed transformer internal carbon trace defect detection model required approximately 8.9 h. Compared with the baseline model, the training time of the improved model increased by approximately 36.9%. At the same time, the proposed model achieved better performance in complex carbon trace defect recognition accuracy and localization of tiny carbon trace defects. Therefore, this increase in training cost is reasonable and acceptable. It should be noted that model training is usually completed offline on a server or high-performance workstation, whereas practical deployment mainly concerns single-frame inference time and resource usage. We further report model parameters, GFLOPs, and inference speed to comprehensively evaluate the engineering application potential of the proposed method in transformer internal carbon trace defect detection.

### 5.3. Analysis of the Impact of Image Enhancement on Defect Detection

In Section 3.2, subjective visual observation and histogram pixel-output comparison preliminarily verified the effectiveness of the proposed Retinex-based image enhancement algorithm with superimposed illumination estimation. To further evaluate the influence of the proposed carbon trace defect image enhancement algorithm on low-light image enhancement, this study adopts more professional quantitative metrics for comprehensive analysis. PSNR, SSIM, VIF, and NIQE are selected to comprehensively evaluate algorithm performance from the perspectives of pixel fidelity, structural preservation, information integrity, and image naturalness.

PSNR reflects the global difference between the enhanced image and the natural illumination reference image; a higher value indicates lower distortion. SSIM (range: 0–1) evaluates the preservation of luminance, contrast, and structural features; the closer the value is to 1, the better the structural information and visual quality of the enhanced image. VIF (range: 0–1) measures the fidelity of visual information transfer; the closer the VIF value is to 1, the less image information is lost during enhancement and the better the enhancement quality. NIQE is a no-reference image quality assessment method that objectively evaluates image quality without requiring an original image; a lower value indicates that the evaluated image is closer to the statistical properties of natural images and has higher quality and naturalness. These objective metrics provide a more comprehensive evaluation of different image enhancement algorithms. The experiment compares the performance differences among MSRCR, DCP algorithms, and the proposed algorithm (ours), as shown in Table 2.

As shown in the table, the proposed image enhancement algorithm achieves significant improvements in the objective evaluation metrics. Compared with DCP-processed images, the PSNR of the proposed algorithm increases from 19.93 to 23.66; in terms of structural feature preservation, SSIM increases from 0.64 to 0.75, VIF increases from 0.89 to 0.92, and NIQE decreases from 0.29 to 0.27. These results indicate that the proposed algorithm has advantages in low-light carbon trace image enhancement. Compared with MSRCR-processed images, the proposed algorithm has lower PSNR and SSIM values, indicating that due to the increased algorithm complexity and dark-detail enhancement, more noise is inevitably introduced into the image.

**Figure 13:**
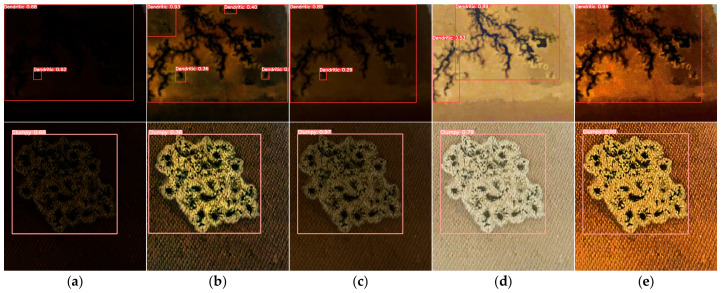
Detection result images: (**a**) original image; (**b**) CLAHE algorithm; (**c**) gamma correction; (**d**) MSRCR algorithm; (**e**) ours.

To further quantitatively evaluate the impact of the proposed algorithm on the detection performance of the recognition model for carbon trace defects on the transformer pressboard, comparative experiments were conducted with multiple mainstream conventional image enhancement algorithms. The aforementioned defect recognition model was applied to perform object detection on the original low-light images and the images enhanced by different algorithms, respectively, with the recognition results presented in Figure 13.

It can be observed from the figure that the illumination condition is the key factor for limiting the detection performance of the pressboard carbon trace defect recognition model, as it severely interferes with the effective extraction of carbon trace features by the detection network. By comparing the recognition results of carbon trace images processed by different enhancement algorithms, the algorithms’ performance differences can be intuitively quantified.

Constrained by low-illumination conditions, the original low-light images yield the worst detection performance, with a significant number of false detections compared to the enhanced images. Although the images enhanced by the CLAHE algorithm achieve improved brightness and contrast, a large amount of noise is introduced, resulting in illumination distortion of the texture features of carbon traces. This makes it difficult for the detection network to effectively identify the defects and severely disrupts the confidence calculation of the detection results. After processing by the gamma correction algorithm, the image brightness is slightly improved and the morphology of carbon traces is more prominent, which facilitates the detection network to capture more detailed features of carbon traces. However, it still fails to effectively extract the fine structure of fine-branched carbon traces. The images enhanced by the MSRCR algorithm exhibit an excessive brightness increase and local overexposure, which leads to over-compression of the contrast of the black texture of carbon traces, loss of partial detailed information, and subsequent false detections.

In contrast, the algorithm proposed in this paper completely retains the key dark-detail information of carbon traces, while effectively enhancing the complex structural features of carbon traces and the target contrast, thus significantly reducing the probability of missed detection and false detection of the detection network. The above results demonstrate that the defect detection performance of images enhanced by the proposed algorithm is significantly superior to that of the original low-light images and those processed by various conventional image enhancement preprocessing algorithms. This algorithm not only realizes effective compensation for low-illumination conditions, but also further strengthens the feature expression of carbon trace defects in the color space, which provides high-quality image data support for the accurate identification and reliable diagnosis of carbon trace defects.

### 5.4. Ablation Experiments

To reduce the influence of random initialization and random training factors, the proposed improved transformer internal carbon trace defect detection model and the baseline model were independently trained five times using different random seeds while keeping the dataset and training hyperparameters consistent. The results were reported in the form of the mean ± standard deviation. The baseline model achieved an average mAP50 of 91.2 ± 0.26%, whereas the improved model achieved 93.9 ± 0.42%, with an average improvement of approximately 2.7%. The baseline model achieved an average mAP50-95 of 70.9 ± 0.15%, whereas the improved model achieved 78.8 ± 0.33%, with an average improvement of approximately 7.9%. It indicates that the proposed improved transformer internal carbon trace defect detection model truly improves carbon trace defect recognition, rather than benefiting from random initialization or training fluctuations.

To further validate the effectiveness of the proposed improved algorithm, ablation experiments were conducted using YOLOv10 as the baseline model with four improved modules. This experimental design quantitatively measures the impact of each modification on performance metrics while demonstrating their contribution to actual improvement. Maintaining consistent data parameters and environmental configurations throughout experimentation, test results on the transformer internal defect discharge carbon trace dataset are presented in Table 3, where ‘+’ indicates the addition of corresponding modules.

As shown in Table 3, the detection performance improved through our proposed enhancements, resulting in stepwise increases in mAP compared to the baseline model. Specifically, adding C2f block integrated with SCSA to the backbone achieved a 1.1% increase in mAP50 and a 2.8% gain in mAP50-95; replacing SPPF with a PKI-ASPP module elevated mAP50 by 1.8% and boosted mAP50-95 by 5.3%; and incorporating DLKA modules improved mAP by 1.6% and enhanced mAP50-95 by 4.7%. The final optimized recognition model of carbon trace in the transformer, featuring improvements to the PKI-ASPP module, deformable large kernel attention module, and C2f-SCSA module, demonstrated significant performance gains over the original architecture: mAP50 increased by 2.7% and mAP50-95 rose by 7.9%. These advancements strengthened the model’s multi-scale feature extraction capabilities, effectively enhancing the precision of the carbon trace detection system.

To verify the effectiveness of the proposed multi-scale feature extraction method, the PKI-ASPP module, for transformer internal carbon trace defect detection, we designed multiple comparison experiments using the SPPF module of the baseline model, the RFB module, the ASPP module, and the proposed PKI-ASPP module. The results are shown in Table 4.

As shown in Table 4, compared with the SPPF module of the baseline model and other multi-scale feature extraction modules, introducing PKI-ASPP achieves a more comprehensive improvement in carbon trace defect detection accuracy. It performs better particularly in precision, recall, mAP, and mAP50-95. Compared with ASPP, these metrics are improved by 0.4%, 0.5%, 0.5%, and 1.4%, respectively, indicating that the module can effectively improve the model’s ability to recognize tiny, low-contrast, and multi-scale carbon trace regions.

In addition, we note that PKI-ASPP introduces a certain number of extra parameters and computation compared with SPPF. Considering that missed carbon trace detection may lead to more serious insulation degradation risks in transformer internal defect detection, slightly reducing inference speed in exchange for higher reliability in detecting complex defect regions has important engineering significance.

**Figure 14:**
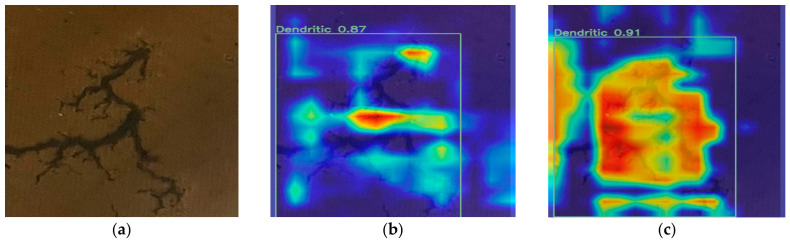
Grad-CAM comparison of detection results with and without DLKA: (**a**) original image; (**b**) without DLKA; (**c**) with DLKA.

To achieve effective fusion of multi-scale differentiated carbon mark features and accurate perception of deep semantic information, this paper embedded the DLKA module into the proposed paperboard defect recognition network. To verify the effectiveness of the proposed scheme, we conducted visual analysis on the feature activation characteristics of the network using Grad-CAM. As shown in Figure 14 below, without the DLKA module, the network exhibited low feature response intensity in partial carbon mark regions, suffered from incomplete activation, and only focused on local features, indicating the model’s insufficient fusion capability for multi-scale carbon mark features. In contrast, with the DLKA module embedded, the feature perception performance of the recognition network is significantly improved.

The heatmap results demonstrate that the feature activation regions can fully cover the carbon mark distribution areas on the paperboard: while accurately focusing on the main trunk of the carbon marks, the module effectively enhances the activation of subtle features at the terminal branches of the carbon marks. The above comparative results fully validate the effectiveness of the DLKA module in perceiving multi-scale differentiated carbon mark features. It can significantly optimize the detection performance of paperboard carbon mark defects.

### 5.5. Real-Time Performance Verification

To further verify the engineering significance of the proposed improved transformer internal carbon trace defect detection model, the optimal weights after training were deployed on the transformer internal inspection robot control terminal. A single carbon trace image was segmented to verify model real-time performance, and the final results are shown in Table 5.

As shown in Table 5, in terms of performance comparison on the robotic equipment, the proposed improved transformer internal carbon trace defect detection model maintains good real-time performance while improving detection accuracy. The model has 12.1 GFLOPs, 5.4 GFLOPs more than the baseline, and 5.3 M parameters, 2.9 M more than the baseline. Due to the introduction of multi-scale feature extraction and attention enhancement structures, the inference time for a single image increases from 0.0107 s to 0.0154 s, and inference speed decreases from 93.5 FPS to 64.9 FPS. Although computational overhead increases, the inference speed of the proposed model remains far above the real-time engineering requirement of more than 30 FPS. It indicates that while improving the reliability of carbon trace defect detection, the model is still feasible for deployment on the transformer internal inspection robot terminal.

### 5.6. Comparative Experiments with Different Models

To further validate the superiority of the proposed multi-scale feature fusion model as a detection method of carbon tracing in the transformer, comparative experiments were conducted against other object detection methods under identical hardware and environmental configurations. The following one-stage models were trained on the same dataset: YOLOv5n, YOLOv7-tiny, YOLOv8n, Gold-YOLO, YOLOv9-tiny, YOLOv10n, YOLOv10s, YOLO11n and ours. Their evaluation metrics are summarized in Table 6.

As demonstrated in the table above, our proposed method exhibits significantly improved detection accuracy compared to other models while maintaining a minimal increase in algorithmic parameters—thus meeting real-time detection requirements. Specifically, mAP surpasses YOLOv5n by 16.9%, YOLOv7-tiny by 16.4%, YOLOv8n by 3.3%, Gold-YOLO by 4.5%, YOLOv9-tiny by 2.3%, Gold-YOLO by 3.5%, YOLOv10n by 2.7%, YOLOv10s by 1.9% and YOLO11n by 2.4%. These results robustly validate the superior performance of our model as a detection method of carbon tracing in the transformer. Regarding real-time capabilities, despite employing a DLKA module that slightly increases parameter count and computational complexity, the model still achieves acceptable inference speed for practical applications. It also indicates potential opportunities for further optimization in architectural lightweighting and computational efficiency.

**Figure 15:**
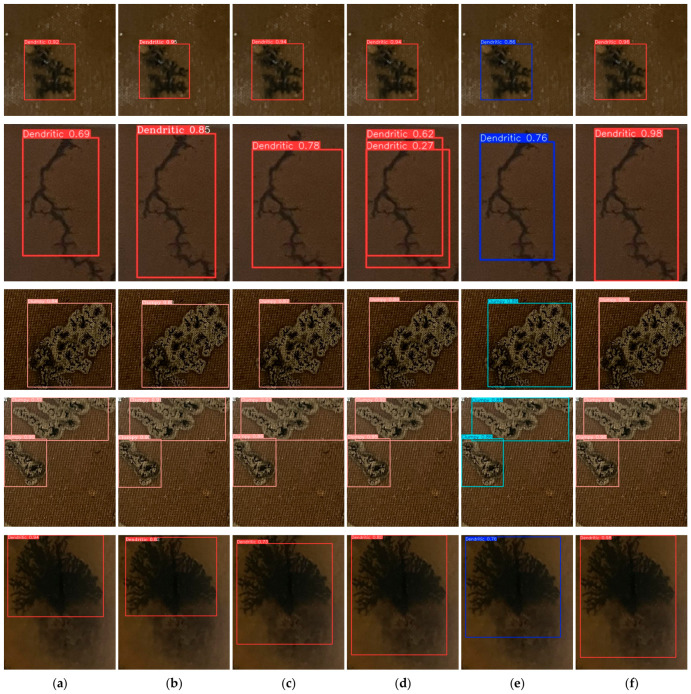
Results of the comparative method experiment: (**a**) Gold-YOLO; (**b**) YOLOv8n; (**c**) YOLOv10n (baseline); (**d**) YOLOv10s; (**e**) YOLOv11n; (**f**) ours.

To further evaluate the proposed model’s performance on detecting complex carbon traces of varying sizes in transformers, we conducted visual comparisons using images selected from the test set of a PD carbon trace dataset, including small-scale dendritic patterns, large-scale dendritic structures, and multi-sized clumpy formations as shown in Figure 15. Compared with Gold-YOLO, YOLOv8n, YOLOv10n, YOLOv10s, and YOLO11n models, our approach achieved superior confidence scores for identifying dendritic carbon traces across all scales. Notably, when detecting large-scale dendritic structures, the predicted bounding boxes demonstrated superior alignment with actual targets in both positioning and dimensions. It indicates effective capture of intricate contours and fine branch characteristics inherent to dendritic patterns at different magnitudes. When processing clumpy carbon traces of varying sizes, our algorithm consistently maintained peak confidence levels. Particularly in scenarios involving multiple clustered targets, the model achieved balanced performance across scales while preserving high confidence and accuracy for each detection. These results demonstrate that the proposed module modifications addressing scale variations play a critical role in enhancing detection capabilities.

## 6. Discussion

Moisture in the transformer internal pressboard, surface contamination, or metallic tips can form discharge carbon traces on the surface of oil-paper insulation. These discharge carbon traces are highly harmful to transformers and, if not handled in time, may lead to insulation breakdown. To avoid large-area power outages caused by insulation breakdown, it is necessary to detect internal insulation discharge carbon traces in transformers and perform timely outage maintenance once carbon traces are found on the pressboard surface. Therefore, this study mainly conducts visual target recognition of clumpy discharge carbon traces on pressboard caused by moisture in the transformer internal insulating pressboard and dendritic carbon traces caused by tips.

The proposed transformer internal carbon trace defect detection model can identify defect regions with obvious visual features in carbon trace images, but its current training labels mainly target two categories: dendritic carbon traces and clumpy carbon traces. Therefore, the model cannot identify insulation breakdown based solely on carbon trace images. Whether insulation breakdown or breakdown risk exists still needs to be comprehensively judged together with electrical tests, PD detection, material performance testing, and operating conditions.

Current intelligent transformer inspection systems have gradually evolved from traditional manual periodic inspection toward the integration of multi-source perception, robotic inspection, online monitoring, intelligent image recognition, digital platforms, and condition assessment models. Typical technical routes include diagnostic methods based on PD signals and online monitoring data, as well as inspection robots for detecting external thermal defects using infrared thermography. Compared with these systems, the proposed method focuses on visible-light image detection of carbon trace defects on the surface of internal transformer insulation materials. It belongs to the category of visible-light-image-based transformer inspection robot methods, but differs in that the robot inspection scenario in this study is internal carbon trace defect detection inside large oil-immersed transformers under oil-filled conditions.

Compared with diagnostic methods based on PD and online monitoring data, the proposed method provides more intuitive image visualization and traceable localization. In recent years, artificial intelligence methods have been widely used for pattern recognition, fault classification, condition prediction, and health assessment based on PD signals and data. These methods can usually reflect internal transformer fault types, discharge activities, or operating-state changes and are important tools for transformer condition assessment. However, they cannot directly display the geometric morphology and precise region of defects on insulation surfaces.

Compared with intelligent diagnosis methods based on infrared thermography, the proposed method differs significantly in detection targets and information sources. Infrared thermal imaging detection mainly relies on surface temperature distribution features of transformers to identify thermal faults such as poor contact, local overheating, bushing abnormalities, and heat-dissipation abnormalities. In recent years, deep learning methods have also been used for denoising, object detection, fault recognition, and semantic segmentation of infrared images of electrical equipment, improving the automation level of thermal defect diagnosis in complex environments [48,49].

Therefore, diagnostic methods based on PD and online monitoring data, infrared diagnostic methods, and the proposed method are complementary in engineering applications. For example, existing substation inspection robots carry visible-light cameras, infrared thermal imagers, ultrasonic sensors, audio acquisition devices, and other equipment to complete tasks such as meter recognition, equipment appearance inspection, infrared temperature measurement, abnormal sound acquisition, and environmental monitoring. If abnormal transformer surface temperature or abnormal discharge signals are detected, and external observation cannot identify the source of fault features, the proposed method can be combined to identify the presence, location, and scale of carbon trace regions on insulation material surfaces inside the transformer body. In summary, the three types of methods provide evidence from different dimensions for transformer condition assessment.

## 7. Conclusions

To facilitate carbon trace detection for oil-immersed transformer internal inspection robots, this paper analyzes the internal image acquisition environment and carbon trace characteristics. To mitigate image degradation caused by insufficient illumination, we propose a Retinex-based image enhancement algorithm with superimposed illumination estimation. Furthermore, we propose an improved model for transformer internal carbon trace detection that achieves significant advancements in two critical aspects: multi-scale target feature capture and complex dendritic feature integration. Comprehensive experimental results demonstrate the following:

1. The proposed image enhancement algorithm, based on Retinex superimposed illumination estimation, conducts HSI color space conversion to decouple intensity from chrominance, effectively correcting color shifts and distortions. Through Laplacian pyramid processing, it extracts the contour characteristics of carbon traces while enhancing high-frequency details. Superimposed illumination images computed via Retinex theory substantially enhance carbon trace contrast. This approach selectively amplifies edge and textural information, which is crucial for distinguishing targets from the background.
2. Integrating the SCSA module into the C2f block enhances multi-scale feature representation by suppressing redundant elements and reinforcing discriminative characteristics. This addresses the limitations of micro-feature extraction while improving complex pattern recognition, thereby obtaining a larger receptive field and more precise multi-scale feature information. Furthermore, replacing the standard SPPF with the PKI-ASPP module prevents the loss of complex morphological information caused by elimination pooling operations. Depthwise separable convolution enables precise deep semantic fusion, leveraging expansive receptive fields to compensate for sparse sampling in dilated convolutions. This resolves critical challenges such as spatial misalignment and fine detail loss during hierarchical feature integration.
3. Integrating the DLKA module into the feature fusion neck network introduces an adaptive and flexible receptive field. This enhances the model’s robustness by enabling the precise feature fusion of irregularly or complexly shaped objects. Consequently, this innovation improves localization accuracy for minuscule defects while reducing false negatives and positives.

Comprehensive experimental results on the PD carbon trace dataset demonstrate that the proposed method exhibits superior performance in handling multi-scale complex objects, meeting both accuracy and real-time requirements for transformer robotic inspection. Our methodology establishes a reliable technical foundation and data support system for future applications, including the preliminary diagnosis of oil-paper insulation surface degradation, monitoring discharge progression, and comprehensive assessment of internal faults and insulation conditions in transformers.

Although the proposed method achieves good detection performance on the current dataset, its generalization capability on larger-scale, multi-source, and cross-equipment datasets still needs further verification. In future work, we plan to collect actual field carbon trace defects and combine them with realistic laboratory reproduction of discharge carbon trace samples and complex environment imaging inside different transformer models to construct a more representative multi-source dataset, thereby improving the generalization capability of the carbon trace detection model in complex operating environments. Second, a scale calibration mechanism will be introduced in subsequent data acquisition and annotation. By combining actual physical size or normalized pixel area, a scale division standard more suitable for transformer internal carbon trace defects will be established. On this basis, evaluation metrics for small-, medium-, and large-scale carbon trace defects can be reported separately to more systematically evaluate multi-scale detection performance. In addition, future work should further conduct online testing on real transformer internal inspection platforms to evaluate model stability under continuous video streams, robot motion disturbance, limited illumination, and embedded real-time inference. This will also facilitate the exploration of fusion mechanisms among detection results, robot autonomous navigation, defect tracking, and multi-view verification.

## Figures and Tables

**Table 1:** Training environment and training parameters.

Training environment	Training platform	Windows 11 x64
	CPU	Intel(R) Core(TM) i5-13400F(2.50 GHz)@32 GB
	GPU	NVIDIA GeForce RTX 4060 Ti@16 GB
	Environment	Python 3.10
	Deep learning framework	PyTorch 2.5.1
	CUDA	12.6
Training parameters	Batch sizes	8
	Lr0	0.01
	Image size	1280 × 1280
	Training epoch	200
	Optimizer	SGD
	Momentum	0.9

**Table 2:** Evaluation metric results of different algorithms.

Algorithms	PSNR	SSIM	VIF	NIQE
MSRCR	27.45	0.79	0.83	0.30
DCP	19.93	0.64	0.89	0.29
Ours	23.66	0.75	0.92	0.27

**Table 3:** Comparison of experimental results of different modules.

Model	P (%)	R (%)	mAP50 (%)	mAP50-95 (%)	Params (M)
YOLOv10n	0.920	0.824	91.2	70.9	2.4
+C2f-SCSA	0.94	0.839	92.3	73.7	2.9
+PKI-ASPP	0.936	0.844	93.0	76.2	3.9
+DLKA	0.932	0.842	92.8	75.6	3.4
Ours	0.948	0.850	93.9	78.8	5.3

**Table 4:** Comparison of detection performance for different multi-scale feature extraction modules.

Model	P (%)	R (%)	mAP50 (%)	mAP50-95 (%)
YOLOv10n (baseline)	0.920	0.824	91.2	70.9
+RFB	0.927	0.833	92.1	73.4
+ASPP	0.932	0.839	92.5	74.8
+PKI-ASPP	0.936	0.844	93.0	76.2

**Table 5:** Real-time performance verification.

Model	Time/s	FPS	GFLOPS	Params/M
YOLOv10 (baseline)	0.0107	93.5	6.7	2.4
Ours	0.0154	64.9	12.1	5.3

**Table 6:** Comparison of performance indexes of each algorithm.

Model	P (%)	R (%)	mAP (%)	Parameters (M)
YOLOv5n	0.843	0.743	77.0	1.8
Yolov7-tiny	0.853	0.752	77.5	6.0
YOLOv8n	0.919	0.830	90.6	3.0
Gold-YOLO	0.893	0.825	89.4	5.6
YOLOv9-tiny	0.912	0.849	91.6	2.0
YOLOv10n (baseline)	0.920	0.824	91.2	2.4
YOLOv10s	0.944	0.838	92.0	8.0
YOLO11n	0.930	0.856	91.5	2.6
Ours	0.948	0.850	93.9	5.3

## Data Availability

The data presented in this study are available on request from the corresponding author. The data are not publicly available due to privacy.

## Abbreviations

The following abbreviations are used in this manuscript:

PD	Partial discharge
DGA	Dissolved gas analysis
NMS	Non-maximum suppression
GUI	Graphical user interface
FPS	Frames per second
HDMI	High-definition multimedia interface
MSRCR	Multi-scale scale Retinex with color restoration
CLAHE	Contrast limited adaptive histogram equalization
DCP	Dark channel prior
